# The Wnt/β-Catenin Pathway Cross-Talks with STAT3 Signaling to Regulate Survival of Retinal Pigment Epithelium Cells

**DOI:** 10.1371/journal.pone.0046892

**Published:** 2012-10-04

**Authors:** Miryam A. Fragoso, Amit K. Patel, Rei E. I. Nakamura, Hyun Yi, Krishna Surapaneni, Abigail S. Hackam

**Affiliations:** Bascom Palmer Eye Institute, University of Miami Miller School of Medicine, Miami, Florida, United States of America; Northwestern University Feinberg School of Medicine, United States of America

## Abstract

Wnt/β-catenin signaling is an essential pathway that regulates numerous cellular processes, including cell survival. The molecular mechanisms contributing to pro-survival Wnt signaling are mostly unknown. Signal transducer and activator of transcription proteins (STATs) are a well-described family of transcription factors. STAT3 induces expression of anti-apoptotic genes in many tissues and is a downstream mediator of protective growth factors and cytokines. In this study, we investigated whether pro-survival Wnt signaling is mediated by STAT3. The Wnt3a ligand activated Wnt signaling in the retinal pigment epithelium ARPE-19 cell line and significantly increased the viability of cells exposed to oxidative stress. Furthermore, Wnt3a increased STAT3 activation and nuclear translocation, as measured by an antibody against phosphorylated STAT3. Reducing STAT3 levels with siRNA eliminated Wnt3a-dependent protection from oxidative stress. Together, these data demonstrate a previously unknown link between Wnt3a-mediated activation of STAT3 and cell survival, and indicate cross-talk between two important pro-survival signaling pathways.

## Introduction

In recent years, numerous cellular processes that are regulated by the Wnt signaling pathway have been identified and characterized, including cellular survival and differentiation, tumorigenesis and stem cell proliferation [1]. The pro-survival activity of the Wnt pathway in the central nervous system (CNS) and other tissues is believed to be mediated by the induction of specific anti-apoptotic genes [2], [3]. Numerous other ligand-receptor mediated signaling pathways are also cytoprotective, but the degree of cross-talk and co-dependence between Wnt signaling and other pro-survival pathways during cellular protection are not well understood.

STATs are a well-described family of transcription factors that are key effectors of a wide variety of cytokines and growth factors, including leukemia inducing factor (LIF), interleukin 6 (IL-6), oncostatin M (OSM) and CNTF [4], [5]. STAT3 regulates cell survival in many tissues by inducing pro-survival genes [4]. Aberrant activation of both STAT3 and Wnt/β-catenin often occurs in malignancies, and the two pathways regulate each other in several cancer cell lines [6], [7], [8]. Interestingly, recent evidence suggests an association between STAT3 and Wnt signaling in non-neoplastic cells. The Wnt ligands Wnt3a, Wnt5a and Wnt6 upregulated STAT3 mRNA and protein in mouse embryonic stem (ES) cells, and LIF synergized with Wnt3a to inhibit ES cell differentiation [9]. Also, Duplin, a negative regulator of the Wnt pathway, binds STAT3 and inhibits its association with DNA in HEK293 cells [10]. However, the interaction between Wnt and STAT3 pathways during cellular injury and protection has not previously been investigated. Therefore, in this study we asked whether Wnt signaling is protective by activating the STAT3 pathway.

Our group and others recently demonstrated that Wnt signaling is increased during neuronal injury in the retina and that it protects retinal neurons and cell lines against various injuries [11], [12], [13], [14], [15], [16]. Retinal pigment epithelium (RPE) cells are an essential cell type positioned in a monolayer between the neuronal cells of the retina and the underlying blood vessels of the choroid, and provide anatomical and functional support for adjacent photoreceptors. Wnt signaling is activated in differentiating RPE and controls expression of the transcription factors *Mitf* and *Otx2* that are essential for RPE development [17], [18]. Aberrant activation of Wnt signaling in adult RPE is associated with pathogenic processes, including RPE migration [19] and expression of inflammatory genes [20]. The role of Wnt signaling in RPE survival during cellular injury is unknown.

STAT3 is expressed in developing and adult RPE and neural retina [21], [22] and is elevated in RPE within pathological choroidal neovascular membranes [23]. Additionally, STAT3 activation was associated with cell-cycle progression in the RPE cell line ARPE-19 [24] and induced neovascularization in the RPE-choroid complex [25]. Despite the importance of STAT3 in the neural retina and RPE, the mechanisms of STAT3 regulation and cross-talk with other signaling pathways have not previously been examined.

In this study, we characterized the role of Wnt signaling in RPE survival in vitro and determined a mechanism of action. Our results show that Wnt3a induced STAT3 activation and translocation into the nucleus. Furthermore, Wnt3a protected cells from oxidative stress, and reducing STAT3 expression eliminated Wnt-dependent cellular survival. Therefore, these findings increase our understanding of the regulation of STAT3 and indicate that Wnt3a is a potential upstream activator of cellular protection in the RPE.

## Results

**Figure 1:**
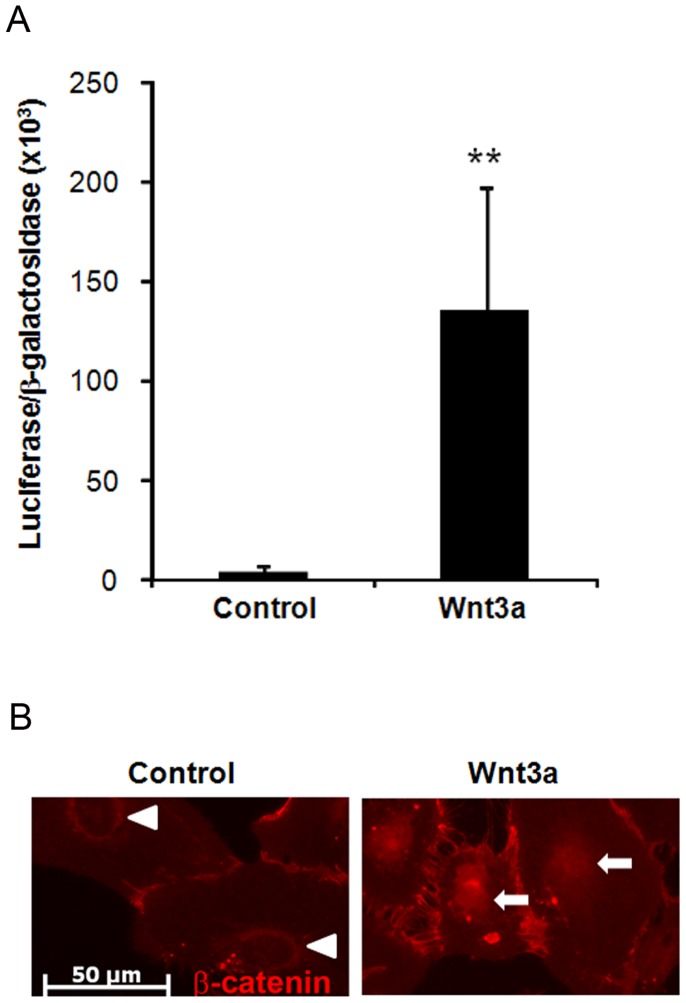
Activation of Wnt signaling in the RPE cell line ARPE-19. (A) Cultured cells were co-transfected with the TOP-FLASH Wnt reporter plasmid and a plasmid containing β-galactosidase, and then incubated with Wnt3a or control conditioned media. Luciferase activity was normalized to β-galactosidase activity. Wnt3a induced a 32-fold increase in luciferase activity compared with control (n = 4, **p = 0.01). (B) Immunodetection of endogenous β-catenin was used to confirm Wnt signaling induction. Cells incubated in control conditioned media have predominantly cytoplasmic and membrane-associated β-catenin (red, arrowheads), indicating low basal levels of Wnt signaling. Stimulation with Wnt3a induced translocation of β-catenin into the nucleus (right, arrows), indicating activation of the Wnt pathway. Scale bar 50 µM.

Activation of the canonical Wnt/β-catenin signaling pathway leads to stabilization and nuclear translocation of β-catenin, followed by binding of the β-catenin protein to the N-terminal domain of TCF/LEF proteins, which induces specific Wnt target genes. The cell type used in this study was the ARPE-19 cell line, which is a widely utilized in vitro model of RPE cells. To confirm that ARPE-19 cells respond to Wnt3a ligand, Wnt signaling was quantified with the TOP-FLASH Wnt reporter luciferase assay, which uses a plasmid containing TCF/LEF binding sequences. Cells treated with control media have levels of luciferase activity that are equivalent to background (Figure 1A). In contrast, incubation with Wnt3a conditioned media increased luciferase activity by 32-fold, indicating activation of Wnt signaling (n = 4, p = 0.01) (Figure 1A).

Nuclear translocation of β-catenin is also a commonly used marker of Wnt signaling [26]. In the absence of Wnt3a, β-catenin is predominantly localized within the cytoplasm and plasma membrane (Figure 1B, left). Upon stimulation of the cells with Wnt3a, β-catenin was translocated into the nucleus (Figure 1B, right), indicating that the RPE cells have the receptors and intracellular components for activating Wnt signaling.

**Figure 2:**
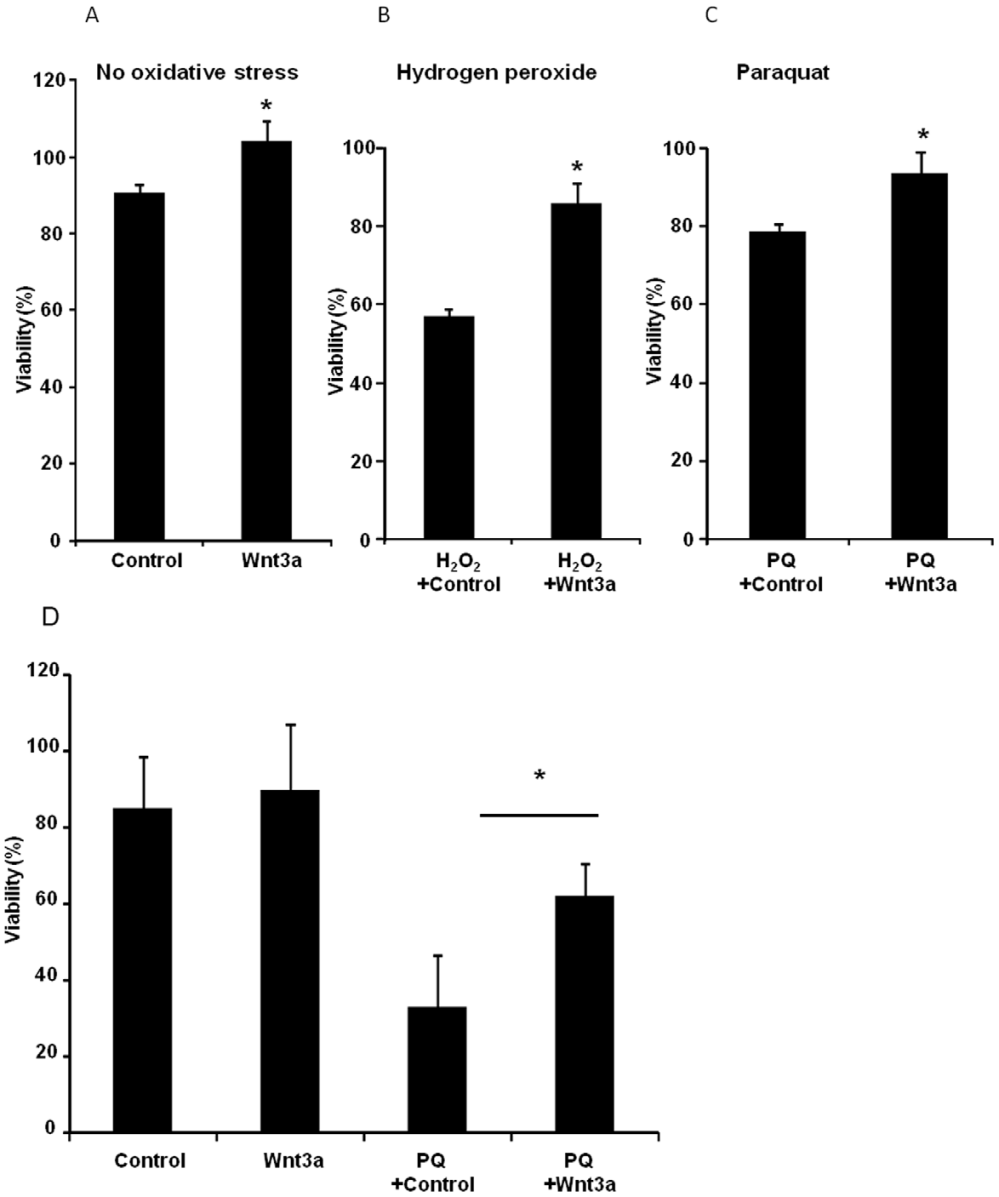
Activation of the Wnt pathway protected ARPE-19 cells from oxidative stress. Cellular viability was significantly increased in the presence of Wnt3a conditioned media compared with control conditioned media when oxidative stress is induced with either 0.4 mM hydrogen peroxide (H_2_O_2_) (n = 4, *p<0.05) (B) or 0.4 mM paraquat (PQ) (n = 9, *p<0.05) (C), both for 24 hr. The viability of uninjured cells was also modestly increased by Wnt3a (n = 9, *p<0.05) (A). Cell viability was also significantly increased by Wnt3a when cells were cultured as polarized post-confluent monolayers on permeable membranes in the presence of 1.6 mM paraquat (n = 3, *p<0.05) (D). Viability was measured by Cell Titer Blue assay.

To determine the role of Wnt signaling on cellular viability, ARPE-19 cells were treated with Wnt3a in the presence and absence of cytotoxic injury. Oxidative stress is a major pathologic factor in numerous diseases. In vitro activators of oxidative stress are hydrogen peroxide (H_2_O_2_) and paraquat (PQ), both of which promote reactive oxygen species production. In the absence of oxidative stress injury, cells treated with Wnt3a conditioned media showed a modest (15%) increase in baseline viability compared with cells treated with control media (n = 9, p<0.05) (Figure 2A). As shown in Figure 2B, Wnt3a protected the cells in the presence of oxidative stress induced by H_2_O_2_, increasing viability by 51% compared with cells that were not treated with Wnt3a (n = 4, p<0.05). Similarly, Wnt3a protected the cells from oxidative stress induced by paraquat, increasing viability by 19% compared with control treated cells (n = 9, p<0.05) (Figure 2C).

The ARPE-19 cells were also cultured as a polarized, post-confluent monolayer on permeable membrane supports, which is more similar to the growth conditions of native RPE cells, and could potentially alter the response of the cells to growth factor stimulation and to H_2_O_2_ injury. Measurements of transepithelial resistance were used to determine when the cells had reached confluency (Figure S1). The cells were treated with paraquat once a resistance of 150 Ω cm^2^ was reached. As shown in Figure 2D, Wnt3a protected ARPE-19 cells that were grown in a polarized confluent monolayer from paraquat-induced oxidative stress, although a higher dose of paraquat was required for toxicity (n = 3, p<0.05) (Figure 2D).

We next investigated whether Wnt3a reduced cell death and/or induced cellular proliferation. Cell death was quantified by immunostaining for propidium iodide and TUNEL, and Western blot detection of caspase 3 and PARP. There were no differences in the number of cells stained with propidium iodide and TUNEL in cultures treated with normal media, Wnt3a or control, in the presence or absence of oxidative stress (Figure S2A–D). Furthermore, the levels of caspase 3 and PARP also showed no difference among treatments (Figure S2E–F), confirming the staining results and indicating that cell death is not a major phenotype in the cultures. The number of proliferating cells in Wnt3a-treated and control-treated cultures were also quantified using calcein staining and Ki67 detection. The calcein staining showed no difference in the number of living cells between treatments. In contrast, quantification of Ki67 staining indicated a modest increase in cell proliferation, by 24%, in the PQ+Wnt3a treatment compared with the PQ+control treatment, although it did not quite reach significance (Figure S2C–D).

Wnt signaling has been reported to induce epithelial to mesenchymal transition (EMT) in numerous cell types [27] and has been implicated in EMT in the RPE [28]. To assess whether Wnt3a signaling induced EMT in the ARPE-19 cells grown under standard culture conditions, within the time-course of our experiments (24 hr), we analyzed the expression of the epithelial marker protein ZO-1 and the mesenchymal marker vimentin, which are commonly used to assess EMT. As shown in Figure S3A–B, there was no change in either marker upon activation of the Wnt pathway, as measured by Western blotting, and their distribution within the cell was also not altered, as measured by immunocytochemistry (data not shown). Therefore, Wnt signaling does not induce EMT in the ARPE-19 cultures within the time-period of our experiments.

**Figure 3:**
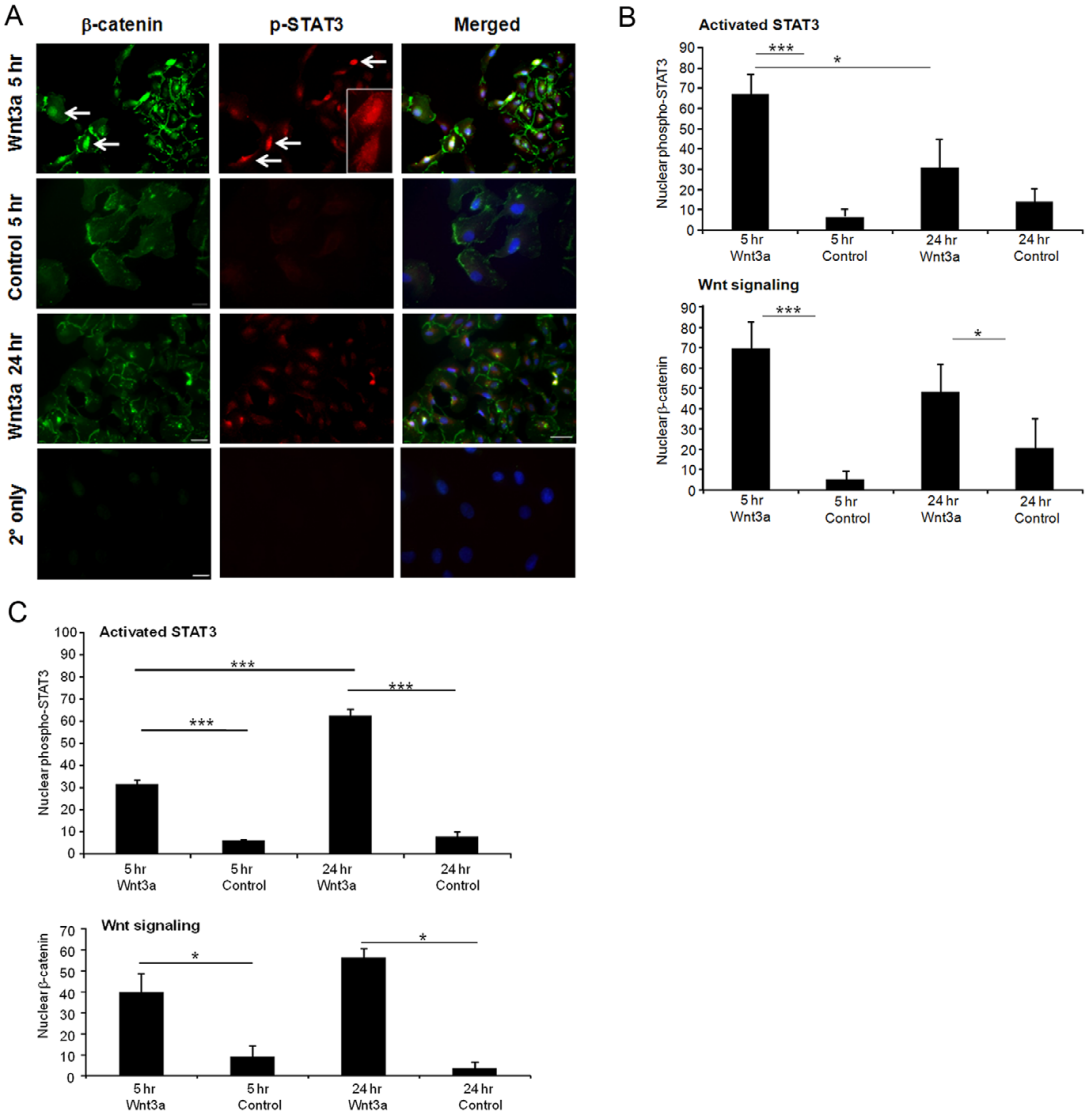
Wnt3a activated STAT3 in ARPE-19 cells. (A) Nuclear localization of phospho-STAT3 is used as a marker of activated STAT3. Incubation of sub-confluent cells with Wnt3a induced transient translocation of pSTAT3 (red) into the nucleus (arrows) after 5 hr incubation, but not after 24 hr. The inset shows a higher magnification. Overlap of pSTAT3 with the DNA dye DAPI (blue) was used to demonstrate nuclear localization. Incubation of cells with control media showed only cytoplasmic phospho-STAT3. Nuclear β-catenin was used as an endogenous marker of Wnt signaling and is observed at both 5 and 24 hr time-points. The absence of primary antibody control (2° only) demonstrates specificity of the immunostaining (see also isotype control antibody in Figure S4). Scale bar in the top 3 rows represents 50 µM; bottom row, 20 µM. (B) Quantification of nuclear phospho-STAT3 and nuclear β-catenin in cells treated with Wnt3a or control for 5 and 24 hr. The percent of total cells with nuclear proteins is shown (n = 3, *p<0.05; ***p<0.001). Wnt3a transiently increased the number of cells with nuclear phospho-STAT3, indicating that Wnt3a activates the STAT3 pathway. (C) Quantification of nuclear pSTAT3 and nuclear β-catenin in cells that were cultured as a polarized post-confluent monolayer, and treated with Wnt3a or control media. Wnt3a induced significant increases in pSTAT3 nuclear localization at both 5 and 24 hrs of treatment (n = 3, *p<0.01). The percent of total cells with nuclear proteins is shown.

Next, we investigated the mechanism by which Wnt signaling increased survival in the presence of oxidative stress. STAT3 is a transcription factor that is activated by specific cytokines and growth factors and regulates apoptosis by inducing pro-survival genes [29], [30], [31]. STAT3 activity requires translocation of the phosphorylated form of STAT3 into the nucleus [5]. Phosphorylation of STAT3 at tyrosine 705 is a commonly used marker of STAT3 activity. Examination of phospho-STAT3 (pSTAT3) with a Tyr705-specific antibody demonstrated translocation of pSTAT3 into the nucleus of the cells after 5 hr incubation with Wnt3a (Figure 3A). Quantification of nuclear pSTAT3 and β-catenin is shown in Figure 3B (n = 3, p<0.001). Cells containing nuclear pSTAT3 costained with nuclear β-catenin, confirming that Wnt signaling activation was associated with STAT3 activation (Figure 3A). It is not known whether the two proteins physically interact in the nucleus. The induction of STAT3 activation by Wnt3a was transient because nuclear pSTAT3 was not significantly increased in cells that were incubated for 24 hr with Wnt3a (Figure 3A, B). In comparison, β-catenin was observed in the nucleus at 5 hr and remained there at 24 hr, which serves as a positive control to confirm Wnt3a activation of the Wnt pathway by the Wnt3a ligand. Control immunostaining with an isotype control antibody, (Figure S4), and omitting the primary antibodies, showed no signal with the same exposure time at either time point, confirming the specificity of the antibodies (Figure 3A, shown for 5 hr). Furthermore, Wnt3a treatment of ARPE-19 cells grown in a polarized, post-confluent monolayer also resulted in a significant level of pSTAT3 activation at 5 hrs that was also evident at 24 hrs (n = 3, p<0.01) (Figure 3C).

**Figure 4:**
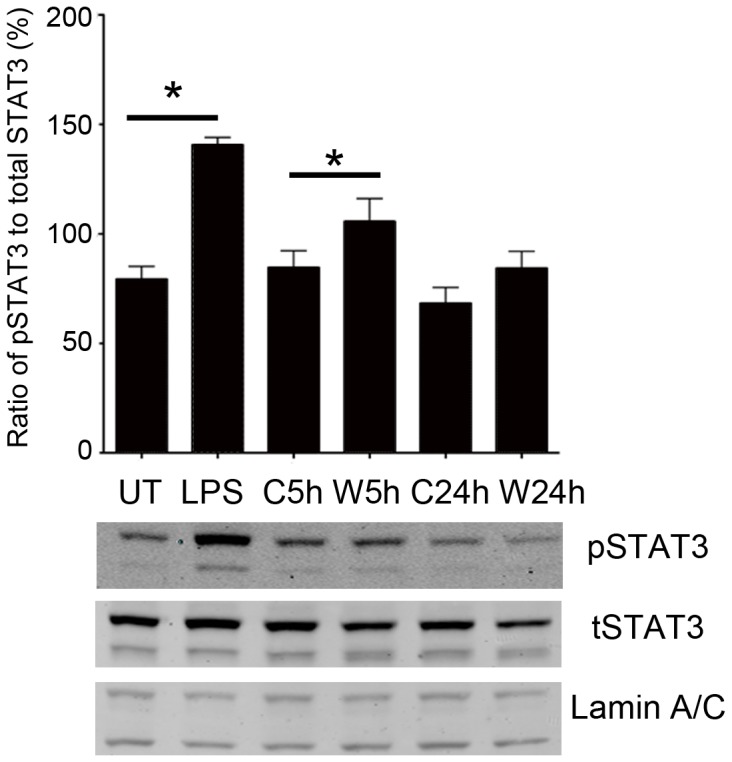
Wnt3a increased levels of nuclear phospho-STAT3. Nuclear fractions were prepared from cells treated with Wnt3a or control for 5 hr (W 5 h, C 5 h), or 24 hr (W 24 h, C 24 h). Lipopolysaccharide (LPS, 100 ng/ml, 5 hr) was used as a positive control, and untreated cells (UT) as the negative control. The blots were probed with antibodies against pSTAT3, total STAT3 (tSTAT3), and the nuclear marker protein lamin A/C. The 5 hr Wnt3a treatment increased the pSTAT3/tSTAT3 ratio by 25% compared with control (n = 3, p = 0.043), confirming that Wnt3a activates STAT3 and induces translocation into the nucleus. Representative blots for each antibody are shown.

Next, Western blot detection of STAT3 was performed on nuclear fractions prepared from cells grown under standard non-polarized conditions and treated with Wnt3a or control. As shown in Figure 4, incubation with Wnt3a for 5 hr increased pSTAT3 by 25% compared with control treatments (n = 3, p = 0.043). In contrast, 24 hr incubation with Wnt did not increase pSTAT3, consistent with the immunostaining results. Therefore, the Western blot results confirm the immunocytochemistry in Figure 3, and support the conclusion that Wnt3a activates STAT3 and induces translocation into the nucleus.

**Figure 5:**
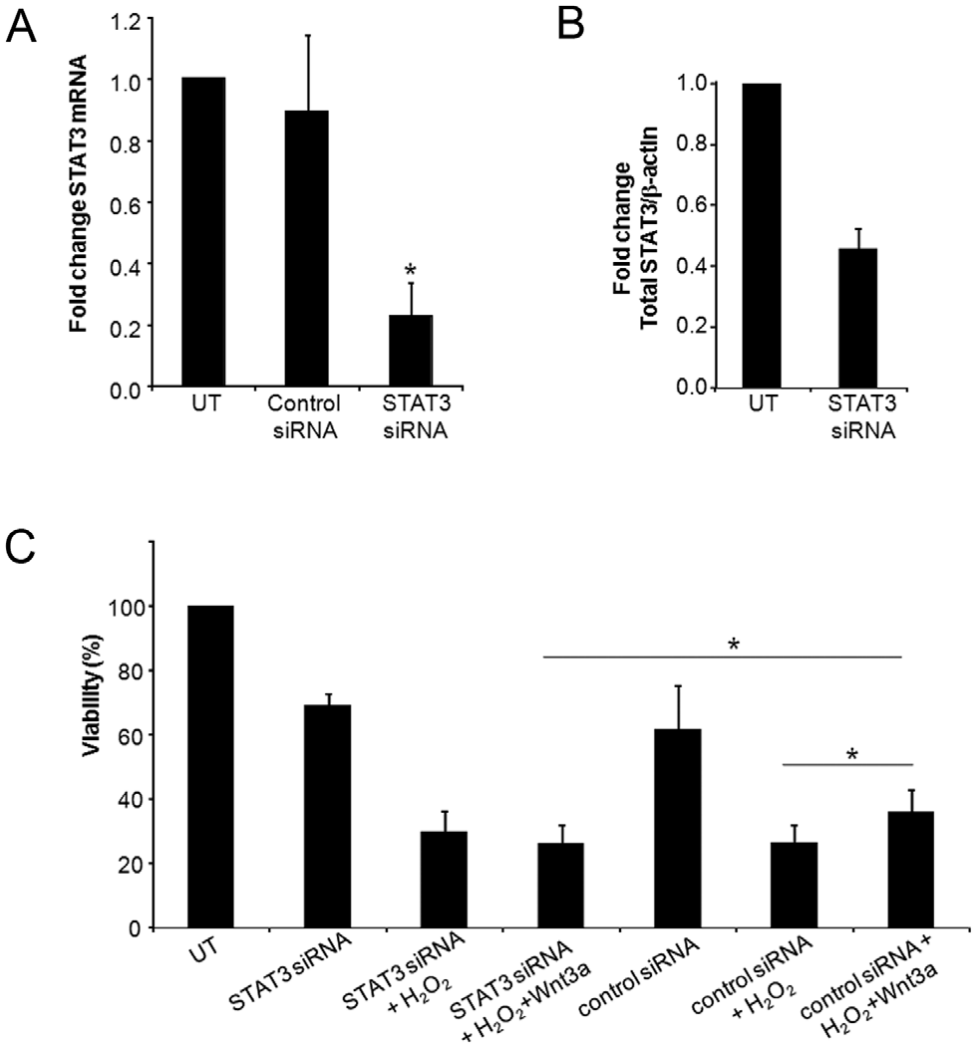
Reducing STAT3 expression eliminated Wnt3a-mediated protection against oxidative stress. (A–B) Efficient reduction of STAT3 expression by siRNA. The cells were transfected with siRNA and STAT3 expression was measured by (A) QPCR (n = 3) and (B) Western blotting (n = 2), and normalized to untransfected cells. *p<0.05. C) The cells were transfected with STAT3 siRNA or a control scrambled siRNA and then were treated with H_2_O_2_ (0.8 mM for 5 hr) with and without Wnt3a. Viability was measured with Cell Titer Blue and normalized to untreated cells (UT). The rescue effect by Wnt3a was eliminated in the presence of STAT3 siRNA but was observed in the presence of control siRNA (n = 3, *p<0.05).

To determine whether STAT3 is required for Wnt3a-mediated protection from oxidative stress, the expression of STAT3 was reduced using specifically targeted siRNA. Transfection of STAT3 siRNA decreased STAT3 mRNA by 77% compared with untransfected cells, and by 75% compared with the scrambled siRNA control, as measured by quantitative PCR (n = 3, p<0.05) (Figure 5A). Similarly, STAT3 siRNA decreased STAT3 protein levels by over 50%, as measured by Western blotting (n = 2) (Figure 5B).

The cells were transfected with either STAT3 siRNA or scrambled control siRNA and then treated with H_2_O_2_ in the presence of Wnt3a. H_2_O_2_ was used to induce oxidative stress because it caused greater cell death than PQ in our previous assays (see Figure 2). As shown in Figure 5C, STAT3 knock-down eliminated Wnt3a mediated-survival, compared with the control siRNA because equivalent viability was observed in STAT3 siRNA + H_2_O_2_ and STAT3 siRNA + H_2_O_2_ + Wnt3a treated cells. In contrast, Wnt3a increased cell viability by 37% in the control siRNA transfections in which STAT3 is present (compare control siRNA + H_2_O_2_ + Wnt3a with control siRNA + H_2_O_2_) (p<0.05). The difference between STAT3 siRNA + H_2_O_2_ + Wnt3a and control siRNA + H_2_O_2_ + Wnt3a is statistically significant (n = 3, p = 0.028). Together, these data indicate a link between Wnt-induced STAT3 activation and the protective effect of Wnt signaling.

## Discussion

The major finding of this study is the identification of a novel molecular mechanism mediating the cytoprotective activity of Wnt signaling. We demonstrated that the pro-survival transcription factor STAT3 is a new effector of Wnt signaling-induced cellular protection of cells exposed to oxidative stress. Wnt3a induced the activation of STAT3, and the protective effect of Wnt3a was eliminated by knock-down of STAT3 using siRNA. Therefore, our findings provide new insight into the regulation of pro-survival pathways during normal and injury conditions.

Our study is the first demonstration of an interaction between Wnt and STAT3 pathways during cellular injury and Wnt-mediated protection. Previous studies on embryonic development and tumor cells support our findings of cross-talk between Wnt/β-catenin and STAT3 pathways. STAT3 expression was upregulated by the Wnt3a, Wnt5a and Wnt6 ligands in cultured mouse embryonic stem cells [9], and by constitutively activate β-catenin in esophageal squamous cell carcinoma [8]. Similarly, total and active STAT3 levels were reduced by down-regulating β-catenin using siRNA in anaplastic large cell lymphoma cells [32]. In contrast, Wnt3a did not increase total or pSTAT3 levels in CML cells [33], indicating cell-type specific interactions between the pathways.

Activation of STAT3 signal transduction is a common route through which certain cytokines and neurotrophic factors mediate cell survival [4], [5]. Evidence from expression analyses and knock-out animals indicates that STAT3 is an important mediator of pro-survival pathways in the retina. The neuroprotective growth factors CNTF, IL6, OSM and LIF induce STAT3 activation and protect against light toxicity and mutation damage [29], [30], [31]. STAT3 cross-talks with other anti-apoptotic pathways, such as AKT, ERK and BDNF/TrkB in various cell types [4], although the interaction between STAT3 and these other pathways has not been examined in the retina. STAT3 has multiple mechanisms of action for protecting cells from oxidative stress, such as associating with the ischemia-induced protective molecule heat shock transcription factor 1 (HSF1) [34], induction of downstream anti-apoptotic genes such as Bcl-2, and by activating Src kinase and upregulating microRNA-21 [35], [36], and these are candidate mechanisms that could be investigated for protection of RPE by Wnt/STAT3.

STAT3 signaling was shown here to be an essential component of Wnt-mediated protection in cultured cells. Although we did not test overexpressed STAT3 in the current study, the STAT3 siRNA approach in Figure 5 supports our conclusion that STAT3 is required for Wnt-mediated protection. Future work will also test whether the combination of Wnt3a and STAT3 overexpression would lead to even greater protection from oxidative stress than Wnt3a by itself. Additional studies will be necessary to determine how the Wnt and STAT3 signaling pathways are linked. For example, Wnt regulation of STAT3 may be transcription-dependent, via a physical interaction between STAT3 and β-catenin or the Wnt-dependent transcription factors TCF/LEF. Alternatively, the interaction between Wnt and STAT3 may be transcription-independent and mediated by Wnt-dependent induction of kinases that activate STAT3. It is also possible that Wnt and STAT3 may function independently. For example, the growth factor CNTF usually acts through STAT3, but in a zebrafish degeneration model, CNTF acted independently of STAT3 and induced protection through MAPK, whereas STAT3 induced Muller glia proliferation [37].

Wnt signaling is protective in numerous cell types, including cancer cells, differentiated retinal cells and progenitor cells, and induces various pro-survival pathways [11], [13], [14], [38], [39]. However, nuclear β-catenin has also been suggested to be a component of proliferative retinal disease by inducing RPE proliferation and migration [19]. Our results indicated that Wnt3a caused a large increase in cellular viability (using an assay that measured mitochondrial function) but only mild increases in proliferation, and no reduction in cell death. Therefore, protection of RPE cells by Wnt/STAT3 signaling may actually have deleterious consequences to the retina by increasing survival of abnormal RPE. Also, the role of Wnt signaling during oxidative stress in RPE cells is controversial. Most studies indicate that Wnt/β-catenin signaling is antagonized by oxidative stress because β-catenin shifts from binding to TCF/LEF proteins to binding to the oxidative-stress induced FOXO transcription factors [40], [41]. While our group and others show that Wnt protected retinal ganglion cells, RPE and photoreceptors against oxidative stress (the present study, [11], [13]), Wnt signaling was recently shown to induce oxidative stress in retinal capillary endothelial cells [42]. Because Wnt signaling often has opposite effects in the same tissue under different contexts [43], further characterizing the interaction between Wnt pathway components and STAT3 during oxidative stress will help clarify the role of Wnt during oxidative injury.

Because both Wnt and STAT3 signaling are activated in the retina [11], [31], our data raises the hypothesis that STAT3 induction may be linked to Wnt signaling in vivo. Wnt regulates survival of other retinal cell types, including photoreceptors [11], [15] and retinal ganglion cells [13], [14] and induces RPE development, angiogenesis and retinal stem cell proliferation [16]. Therefore, future studies will test the possibility that STAT3 acts as a common effector of additional Wnt-dependent activities in the retina. Similarly, STAT3 also negatively regulates differentiation of rod photoreceptors from progenitor cells [21] and promotes glial differentiation [44], which are also activities mediated by Wnt signaling [45], [46], raising the question of whether STAT3 regulates these phenotypes in concert with Wnt ligands.

Our findings in the RPE cell line allow the generation of a testable hypothesis that will investigate whether there is an association between Wnt and STAT3 in vivo. The ARPE-19 cells used in this study are widely utilized as a model of human RPE that exhibit lower variability than primary RPE cultures and posses RPE-like properties, including polarization, tight junction formation, phagocytosis of rod outer segments and immunologic responses [47], [48], [49], and are often used to study regulation of RPE survival [50], [51]. As with all cell lines, certain properties of ARPE-19 vary with culture conditions, and microarray analysis has shown gene expression differences between ARPE-19 and native RPE [52], [53]. Post-confluent ARPE-19 cells required more paraquat for equivalent toxicity as subconfluent cells, as reported in other studies, possibly due to density-dependent changes in mitochondrial-specific protein expression or turnover, such as mitochondrial MnSOD [54], or the formation of tight junctions in confluent cultures that allows transfer of protective factors, and/or growth arrest at confluency that reduces apoptosis susceptibility [55], [56]. However, the major findings in this study are the same for subconfluent and postconfluent cells. Additionally, ARPE-19 cells do not have melanin, which can function as an antioxidant [57], [58], indicating that the Wnt-STAT3 pathway is just one of multiple protective pathways that could be active in vivo. Although Wnt and STAT3 have only been linked in a retina-derived cell line thus far, the importance of both STAT3 and Wnt pathways is already established in the retina. Therefore, defining the Wnt/STAT relationship in RPE in vitro during acute oxidative stress provides an important starting point towards identifying the role of STAT3 and Wnt interactions in vivo and the effect of Wnt/STAT3 cross-talk in RPE during disease under chronic oxidative stress conditions.

## Materials and Methods

### Cell Culture and Reagents

The RPE cell line ARPE-19 is derived from adult human retinal pigment epithelium and is a non-transformed cell line that exhibits many properties characteristic of differentiated RPE in vivo [47]. ARPE-19 cells were purchased from ATCC (Manassas, VA) and all experiments were performed in early passage cultures with consistent densities (>95% density) to minimize assay variability. The cells were maintained at 37°C, 5% CO_2_ in Dulbecco’s modified Eagle’s medium/F12 (Invitrogen Carlsbad, CA) and supplemented with 10% heat inactivated fetal bovine serum (FBS), 100 U/ml of penicillin and 100 µg/ml of streptomycin. Cells grown under non-polarized subconfluent conditions were passaged every 3–4 days, and cells grown to polarized post-confluent cultures were first seeded on uncoated Transwell Permeable supports (0.40 µm membrane, Costar) and cultured until a resistance of 150 Ω cm^2^ was achieved, as measured by an electrical resistance meter. Wnt3a conditioned media was prepared from mouse L-cells stably expressing Wnt3a (ATCC, Manassas VA) and control conditioned media without Wnt3a was prepared from parental L-cells. Both conditioned media were filtered and mixed 1∶1 with normal growth media prior to use [26]. All the experiments used conditioned media from the same preparation batch.

### Wnt Signaling Luciferase Reporter Assays

Cells were cotransfected with a 4∶1 ratio of the TOP-FLASH luciferase reporter plasmid (generously provided by Dr. R. Moon, HHMI, University of Washington) and a LacZ containing plasmid, using lipofectamine2000 (Invitrogen, Carlsbad CA), as described in [59]. The mutated FOP-FLASH plasmid was used as a negative control (not shown). Transfected cells were incubated with Wnt3a or control conditioned media for 24 hr and the cells were harvested and lysed in Reporter lysis buffer (Promega, Madison, WI). Luciferase activity was measured in a Lumistar Galaxy luminometer (BMG Labtech, Offenburg, Germany) and normalized to β-galactosidase activity.

### Immunohistochemistry

Cells were plated onto glass cover-slips in normal growth medium and allowed to attach overnight. For polarized post-confluent monolayers, the cells were seeded on Transwell Permeable supports (0.40 µm membrane, Costar) and cultured until a resistance of 150 Ω cm^2^ was achieved. The cells were either left untreated or were treated with Wnt3a or control conditioned media for 5 or 24 hr, and then fixed in 4% PFA in PBS for 20 minutes at room temperature. The cells were then permeabilized with 0.3% Triton X-100 in PBS for 10 minutes, blocked with 10% normal goat serum (NGS) in PBS for 1 hour at room temperature and then incubated with mouse anti-β-catenin (1∶100 dilution, BD Transduction Laboratories, San Jose, CA), rabbit anti-phospho-STAT3 (1∶100 dilution, Cell Signaling Technology, Danvers, MA), rabbit anti-ZO-1 (1∶100 dilution, Santa Cruz), or normal rabbit IgG control (Santa Cruz), or mouse anti-vimentin (1∶200 dilution, Sigma) diluted in 2% NGS/0.03% Triton X-100 in PBS, overnight at 4°C. The coverslips and membranes were washed several times in 0.03% Triton X-100 in PBS, then were incubated with the corresponding Alexa 546 or Alexa 488 goat anti-mouse or anti-rabbit IgG secondary antibodies (1∶500, Molecular Probes-Invitrogen, Carlsbad, CA) for 1 hr at room temperature, followed by additional washing and then counterstaining with 4′,6-diamidino-2-phenylindole (DAPI). The cover-slips and membranes were viewed using a Zeiss fluorescent microscope (Axiovert 200; Carl Zeiss, Oberkochen, Germany) and images were obtained with a digital camera (Axiocam Zeiss). The microscopy and photographic settings were kept constant for samples and controls. Cells with nuclear localized β-catenin and phospho-STAT3 were counted in five fields (100–300 cells per field) for each experiment. Many of cells had both nuclear and cytoplasmic STAT3, and because they had some portion of STAT3 in the nucleus they were counted as having activated STAT3. The percentage of such cells was calculated as a proportion of total cells.

### Viability Assays

Cell viability was measured using the Cell Titer Blue assay (Promega, Madison, WI). The cells were seeded into 96-well plates and triplicate wells were treated with hydrogen peroxide (H_2_O_2_) (0.4 mM for 24 hr or 0.8 mM for 5 hr) or paraquat (0.4 mM for 24 hr) (Sigma, St. Louis, MO) to induce oxidative stress, in the presence of Wnt3a conditioned media or control conditioned media lacking Wnt3a. The percent viability was calculated by normalizing to untreated cells. Cells were also seeded and cultured on 12 mm Transwell Permeable supports (0.40 µm membrane, Costar, Corning, NY) and cultured until a resistance of ≥150 Ω cm^2^ was achieved and were then treated with 1.6 mM paraquat for 24 hours to induce oxidative stress in the presence of normal growth media, Wnt3a conditioned media or control conditioned media.

### Quantitative PCR Analysis

Total RNA was isolated using TRIzol (Invitrogen, Carlsbad, CA), according to the manufacturer’s instructions, and cDNA was synthesized using Thermoscript (Invitrogen Carlsbad, CA) from 1 µg of total RNA, as described previously [60]. All primers were designed to cross at least one intron and were specific to the gene of interest. Primers that recognize the housekeeping gene acidic ribosomal phosphoprotein (ARP) were used as a normalization control [60]. The primers are: ARP: sense 5′-ATCTGCTGCATCTGCTTG-3′; antisense 5′-CGACCTGGAAGTCCAACTAC-3′; STAT3: sense 5′ ACAGATTGCCTGCATTG-3′; antisense 5′-CTGCTAATGACGTTATCCAGT-3′. Quantitative PCR analysis was performed using SYBR green (Biorad, Hercules CA) with the Mastercycler Real-time PCR system (Eppendorf, Haupaugge, NY). Relative transcript levels of each gene were calculated using the delta-delta Ct method [61].

### STAT3 siRNA Knock-down

STAT3 expression was reduced by siRNA molecules using the protocol and reagents in the Silencer siRNA Starter Kit (Ambion). Two STAT3 siRNA molecules were tested by QPCR, and the most effective molecule was used in subsequent studies (sense 5′-GAGUUGAAUUAUCAGCUUA-3′, anti-sense 5′- UAAGCUGAUAAUUCAACUC-3′). A scrambled siRNA was used as the negative control. The cells were plated in 6-well plates at a density of 1.20×10^5^ per well and in 96-well plates at a density of 6×10^3^ per well, and were transfected with the siRNA molecules using the siPORT reagent (Ambion, Austin, TX), according to the manufacturer’s protocol. At 48 hr post-transfection the cells were treated with H_2_O_2_ and Wnt3a, then assayed 24 hr later for viability, or harvested for QPCR analysis.

### Western Blotting

ARPE-19 cells (5×10^5^/well) were treated with Wnt3a or control, for 5 or 24 hr, harvested by centrifugation, and cellular fractions were prepared using the Ne-PER Nuclear and Cytoplasmic Extraction Reagent (Pierce), according to the manufacturer’s directions. Five micrograms of protein were resolved on 4-12% Bis-Tris acrylamide precast gels with MOPS buffer, and the proteins were then transferred onto polyvinylidene fluoride (PVDF) membranes. The blots were probed using antibodies that detect pSTAT3 (Cell Signaling Technology), total STAT3 (Cell Signaling Technology), and lamin A/C (Cell Signaling Technology), followed by horseradish peroxidase (HRP)-conjugated secondary antibodies (Santa Cruz Biotechnology Inc, Santa Cruz CA). The Li-Cor Odyssey system was used for the development and quantification of bands. For the EMT analysis, caspase 3 and PARP Western blots, the cells were treated as above and whole cell lysates were prepared in lysis buffer containing a proteinase and phosphatase inhibitor cocktail (50 mM Tris, pH7.4, 150 mM NaCl, 1% NP40, 0.05% SDS). Twenty micrograms of total protein were resolved in 4–12% SDS–PAGE gels using Tris-glycine buffer and the proteins were then transferred onto polyvinylidene fluoride (PVDF) membranes and probed using the antibodies that detect total ZO-1 (Santa Cruz Biotechnology Inc, Santa Cruz CA), vimentin (Sigma Aldrich), PARP (Sigma), caspase-3 (Cell Signaling) and β-actin (Sigma), and detected as above. Bands were quantified using NIH Image J. The values were normalized to PARP or β-actin to correct for loading differences.

### Statistical Analysis

Statistical analysis of the data was performed by Student’s *t*-test or two-way analysis of variance (ANOVA) using GraphPad Prizm. For the siRNA viability assays, the differences between groups were evaluated by two-sample *t*-test using SPSS.

## Supporting Information

Figure S1 **Resistance measurements of polarized post-confluent ARPE-19 cells.** ARPE-19 cells were seeded on Transwell permeable membranes (250,000 cells per well) and were cultured for up to 9 days. The baseline resistance of the transwells when the seeding cells are absent (media only) was 102±2.6 Ωcm^2^. Transwell resistance measurements were taken every few days and resistance reached maximum on day 4 and remained above that level (n = 3). Baseline-subtracted readings are shown; the maximum resistance after subtracting the baseline was 53+1.0 Ωcm^2^, which is consistent with other studies on ARPE-19 cells.
(TIF)

Figure S2 **Investigating the effect of Wnt3a on ARPE-19 proliferation and death in the presence and absence of oxidative stress.** ARPE-19 cells were treated with normal (UT), Wnt3a, or control media in the presence or absence of oxidative stress (0.8 mM paraquat). Cells were immunolabeled for markers of cell death (propidium iodide (PI) and TUNEL) and proliferation (Ki67 and calcein), and DAPI and Hoechst staining were used to identify the cell nuclei. (A–D) There was no significant change in percentage of positive cells staining for PI (n = 3, 0% of the cells in each treatment were PI-positive), calcein (n = 3, 100% of the cells in each treatment were calcein-positive) or TUNEL (n = 3, 0% of the cells in each treatment were TUNEL-positive). There was a small increase in Ki67 positive cells in the PQ+Wnt3a treatment compared with PQ+Control (n = 3, not significant). (E,F) Western blots for caspase 3 and PARP also showed no significant difference amongst treatments.
(TIF)

Figure S3 **Wnt3a treated cells do not undergo epithelial to mesenchymal transition (EMT).** Cells were treated Wnt3a or control media for 5 hrs or 24 hrs followed by measurements of the EMT markers ZO-1 and vimentin. (A,B) The cells did not show any change in either proteins by Western blotting (n = 3), indicating no change in the epithelial phenotype. Mouse eye lysate was used as a positive control.
(TIF)

Figure S4 **Immunostaining controls for the anti-phospho-STAT3 primary antibody in Wnt3a-treated cells.** Lack of detection of STAT3 with the isotype control antibody (top) or no primary antibody (bottom).
(TIF)

Methods S1 (DOCX)
