# Supplementary figures and images for: The Wnt/β-Catenin Pathway Cross-Talks with STAT3 Signaling to Regulate Survival of Retinal Pigment Epithelium Cells
Source: PLoS One. 2012 Oct 4;7(10):e46892. doi: 10.1371/journal.pone.0046892 (PMC3464242; doi:10.1371/journal.pone.0046892)

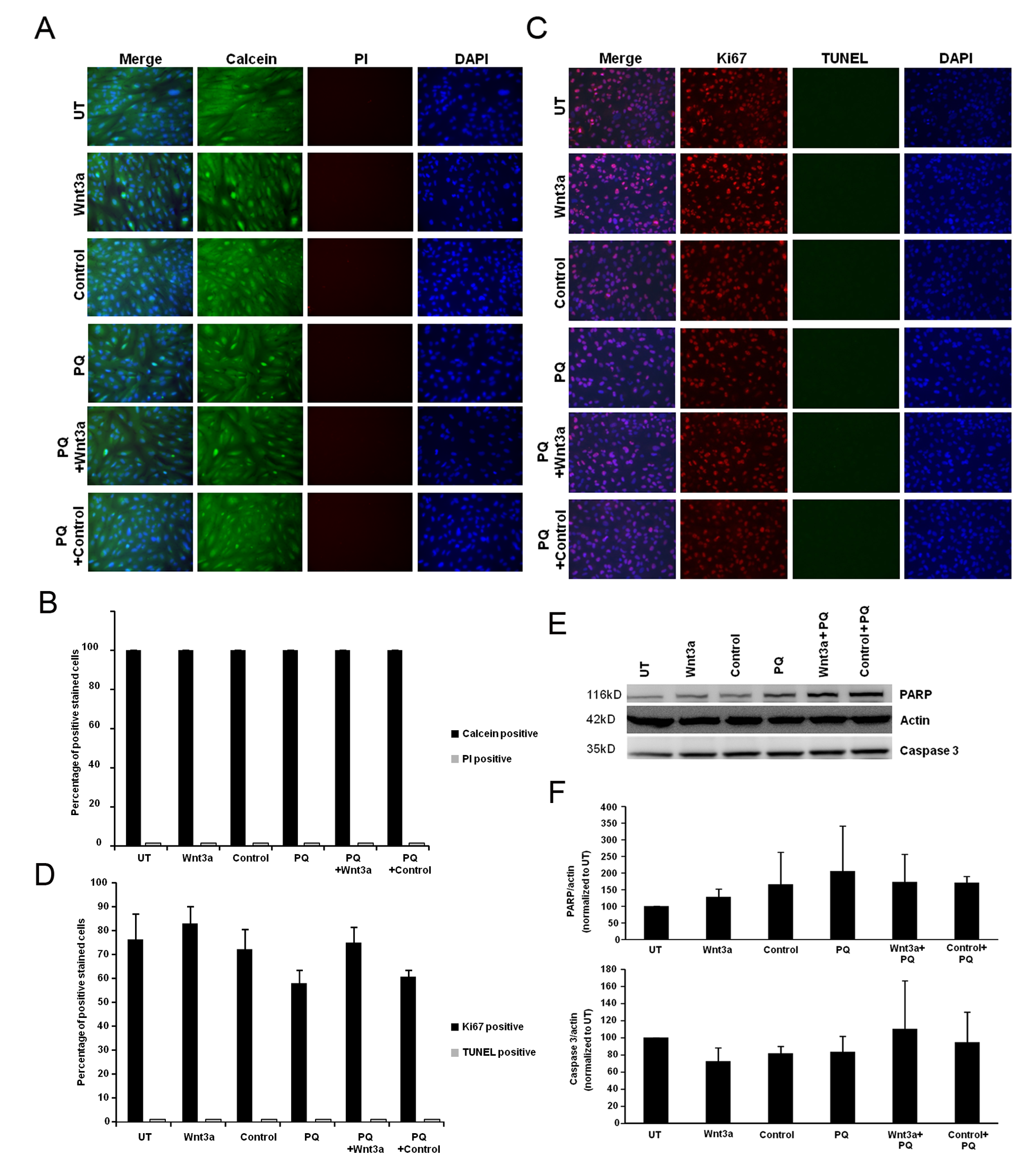

Supplement: Figure S2 — Investigating the effect of Wnt3a on ARPE-19 proliferation and death in the presence and absence of oxidative stress. ARPE-19 cells were treated with normal (UT), Wnt3a, or control media in the presence or absence of oxidative stress (0.8 mM paraquat). Cells were immunolabeled for markers of cell death (propidium iodide (PI) and TUNEL) and proliferation (Ki67 and calcein), and DAPI and Hoechst staining were used to identify the cell nuclei. (A–D) There was no significant change in percentage of positive cells staining for PI (n = 3, 0% of the cells in each treatment were PI-positive), calcein (n = 3, 100% of the cells in each treatment were calcein-positive) or TUNEL (n = 3, 0% of the cells in each treatment were TUNEL-positive). There was a small increase in Ki67 positive cells in the PQ+Wnt3a treatment compared with PQ+Control (n = 3, not significant). (E,F) Western blots for caspase 3 and PARP also showed no significant difference amongst treatments. (TIF) [file pone.0046892.s002.tif]

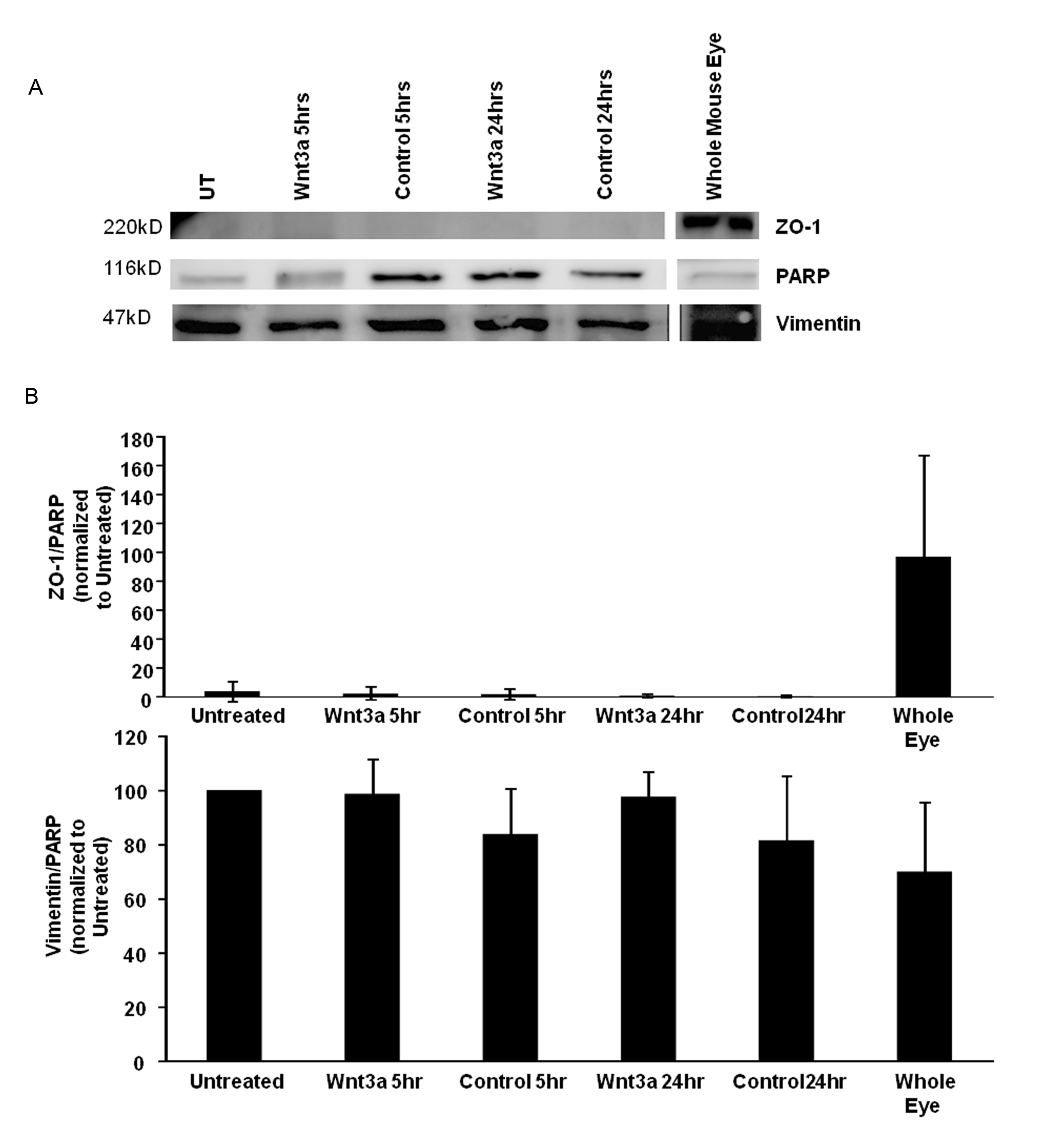

Supplement: Figure S3 — Wnt3a treated cells do not undergo epithelial to mesenchymal transition (EMT). Cells were treated Wnt3a or control media for 5 hrs or 24 hrs followed by measurements of the EMT markers ZO-1 and vimentin. (A,B) The cells did not show any change in either proteins by Western blotting (n = 3), indicating no change in the epithelial phenotype. Mouse eye lysate was used as a positive control. (TIF) [file pone.0046892.s003.tif]

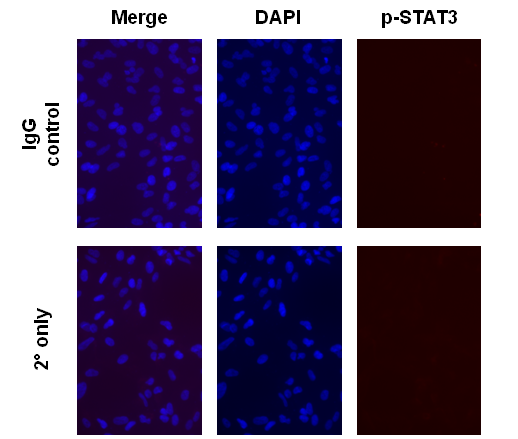

Supplement: Figure S4 — Immunostaining controls for the anti-phospho-STAT3 primary antibody in Wnt3a-treated cells. Lack of detection of STAT3 with the isotype control antibody (top) or no primary antibody (bottom). (TIF) [file pone.0046892.s004.tif]
