# Supplementary material for: The Wnt/β-Catenin Pathway Cross-Talks with STAT3 Signaling to Regulate Survival of Retinal Pigment Epithelium Cells
Source: PLoS One. 2012 Oct 4;7(10):e46892. doi: 10.1371/journal.pone.0046892 (PMC3464242; doi:10.1371/journal.pone.0046892)
Supplement: Methods S1 — (DOCX) [file pone.0046892.s005.docx]

Supplemental Methods.

Live-Dead Assay

Cells were cultured in 6-well plates and treated with normal growth media, Wnt3a or control media in the presence or absence of 0.8 mM paraquat for 24 hrs. Calcein-AM (1 µM, Invitrogen, Carlsbad, CA) was used to assess cell survival, Hoechst dye (1:5000, Invitrogen) was used as a nuclear marker, and propidium Iodide (1:5000; Invitrogen) was used to measure dead cells. Images were taken of random fields using a Zeiss fluorescence microscope (Axiovert 200; Carl Zeiss, Oberkochen, Germany) and analyzed. Labeled cells were counted in five to ten fields for each experiment (100–300 cells per field).

TUNEL

Cells treated with normal growth media, Wnt3a or control media, in the presence or absensce of 0.8mM paraquat. The cells were then fixed with 4% paraformaldehyde (PFA) in PBS for 20 min at room temperature, permeabilized with 0.3% Triton X-100 for 10 min, and apoptosis induction was quantified by the TUNEL assay using the ApoTag Fluorescein InSitu Apoptosis detection Kit (Chemicon, Temecula, CA) according to the manufacturer’s instructions. Cells were also stained with Ki67 (1:100 dilution, Abcam 155580) as a proliferation marker. After counterstaining with 40,6-diamidino-2-phenylindole (DAPI), images were taken of random fields using a Zeiss fluorescence microscope (Axiovert 200; Carl Zeiss, Oberkochen, Germany) and analyzed. Cells were counted in five to ten fields for each experiment (100–300 cells per field). The number of TUNEL-positive and Ki67-positive cells and the total number of cells present (DAPI positive cells) were counted and the percentage of staining cells was calculated for each treatment.
